# Molecular insights and diagnostic advances in strawberry-infecting viruses

**DOI:** 10.3389/fmicb.2025.1655696

**Published:** 2025-08-06

**Authors:** Bo Yan, Mengjiao Lu, Jijing Han, Yuhao Cao, Fei Yan, Xuemei Song

**Affiliations:** State Key Laboratory for Quality and Safety of Agro-products, Key Laboratory of Biotechnology in Plant Protection of MARA, Key Laboratory of Green Plant Protection of Zhejiang Province, Ningbo University, Ningbo, China

**Keywords:** strawberry, virus, molecular biology, mixed infections, detection technology

## Abstract

Strawberry (*Fragaria* × *ananassa* Duch.) production is threatened by more than 20 viral pathogens, which frequently occur in mixed infections, leading to significant yield losses and diagnostic complexities. This review summarizes recent advances in the biology and molecular biology of the major strawberry-infecting viruses, strawberry vein banding virus, strawberry necrotic shock virus, strawberry mottle virus, strawberry latent ringspot virus, strawberry mild yellow edge virus, strawberry pallidosis-associated virus, and strawberry polerovirus 1. It reviews the molecular interactions between viruses and strawberry and also highlights cutting-edge detection technologies, including high-throughput sequencing, RT-PCR/qPCR, and isothermal amplification coupled with lateral flow assays. Despite these advances, critical research gaps remain, particularly in the functional characterization of viral proteins, the mechanisms underlying synergistic and antagonistic interactions in mixed infections, and the development of rapid, field-deployable diagnostic tools. Addressing these challenges is essential for enhancing virus-free certification programs, guiding targeted breeding efforts, and implementing effective disease management strategies to ensure the sustainability of global strawberry production.

## 1 Introduction

Strawberry (*Fragaria* × *ananassa* Duch.) is one of the most economically important berry crops cultivated worldwide for its high nutritional value and consumer appeal. However, strawberry production is highly vulnerable to various viral pathogens that significantly compromise plant vigor, fruit yield, and quality. More than 20 viruses have been reported to infect strawberry plants often causing non-specific symptoms such as stunting, leaf distortion, chlorosis, and reduced fruit size, making field diagnosis unreliable. Some of the viruses do not apparently cause symptoms. Mixed infections are common in both commercial cultivars and wild populations, further complicating disease management strategies (Li and Yang, 2011; Silva-Rosales et al., 2013). The increasing use of vegetative propagation and the global trade of nursery stocks have facilitated the dissemination of these viruses across regions and continents, raising serious phytosanitary concerns.

Over the past decade our understanding of strawberry-infecting virus molecular biology (including virus discovery, genomic architecture, viral protein functions, and virus-host protein interactions) has advanced significantly. As a result there is a robust theoretical foundation for deciphering viral pathogenic mechanisms, delineating virus-host interplay, and understanding the evolutionary arms race between viruses and strawberry. While substantial progress has been made, further research is needed to fully elucidate the complex mechanisms underlying these interactions.

Breakthroughs in molecular diagnostics have also transformed strawberry-infecting virus detection and characterization. Cutting-edge technologies—particularly high-throughput sequencing (HTS), reverse transcription PCR (RT-PCR), and CRISPR-based assays—have dramatically improved detection sensitivity and specificity (Medberry et al., 2023). These innovations not only facilitate the identification of known and emerging viruses but also support large-scale epidemiological investigations. Furthermore, they play a pivotal role in certification programs aimed at producing virus-free planting materials, thereby safeguarding strawberry production.

This review examines recent progress in understanding viral protein functions and virus-host interactions among the major strawberry-infecting viruses. We review cutting-edge developments in viral detection technologies and evaluate their applications for disease diagnosis and management in strawberry production. Finally, we identify critical knowledge gaps and propose key research directions to advance the field of strawberry virology.

## 2 Biological and molecular biological characteristics of the main strawberry-infecting viruses

**Figure 1:**
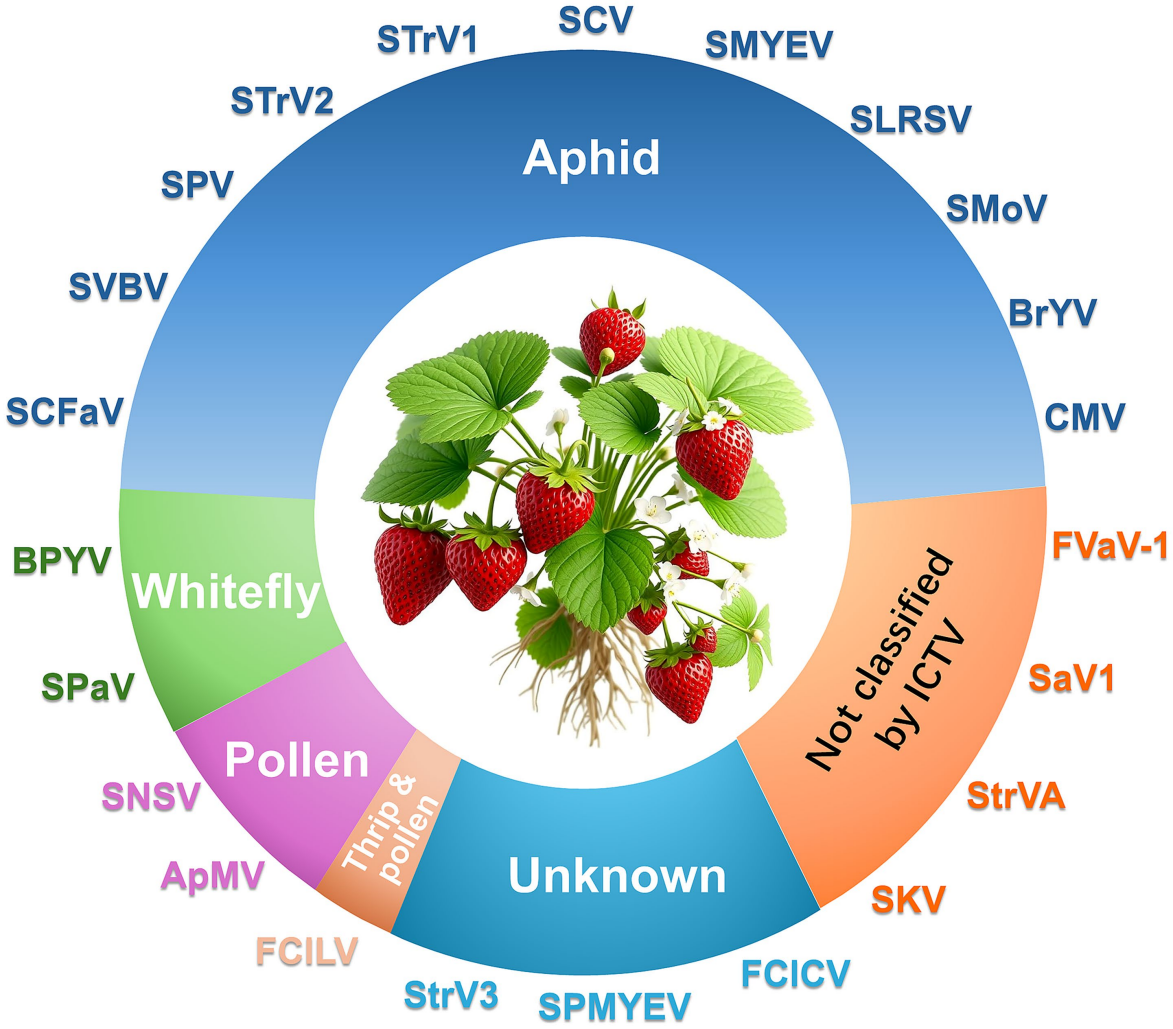
Major viruses infecting strawberry and their transmission modes.

More than 20 viruses are known to infect strawberry, some of which are strawberry-specific (e. g., SVBV, SNSV, and SMoV) while others are of non-strawberry origin (e.g., cucumber mosaic virus, CMV; beet pseudo yellows virus, BPYV; and brassica yellows virus, BrYV) (Figure 1; Table 1). Additionally, several novel viruses recently identified in strawberry await formal classification by the ICTV (Table 2). We here focus on seven agriculturally significant strawberry-specific viruses, synthesizing current knowledge of their biological properties, molecular mechanisms, and detection challenges.

**Table 1:** Viruses infecting strawberry that are officially classified by ICTV.

Virus name	Virus abbreviations	Species name	Family	Transmission
Strawberry pallidosis-associated virus	SPaV	*Crinivirus palidofragariae*	*Closteroviridae*	Whitefly
Beet pseudoyellows virus	BPYV	*Crinivirus pseudobetae*	*Closteroviridae*	Whitefly
Strawberry chlorotic fleck-associated virus	SCFaV	*Closterovirus fragariae*	*Closteroviridae*	Aphid
Strawberry vein banding virus	SVBV	*Caulimovirus venafragariae*	*Caulimoviridae*	Aphid
Strawberry polerovirus 1	SPV	*Polerovirus SPV*	*Solemoviridae*	Aphid
Strawberry virus 3	StrV3	*Deltanucleorhabdovirus fragariae*	*Rhabdoviridae*	Not by aphids
Strawberry virus 2	STrV2	*Alphacytorhabdovirus betafragariae*	*Rhabdoviridae*	Aphid
Strawberry virus 1	STrV1	*Alphacytorhabdovirus alphafragariae*	*Rhabdoviridae*	Aphid
Strawberry crinkle virus	SCV	*Alphacytorhabdovirus fragariarugosus*	*Rhabdoviridae*	Aphid
Strawberry mild yellow edge virus	SMYEV	*Potexvirus fragariae*	*Alphaflexiviridae*	Aphid
Strawberry latent ringspot virus	SLRSV	*Stralarivirus fragariae*	*Secoviridae*	Aphid
Strawberry mottle virus	SMoV	*Sadwavirus fragariae*	*Secoviridae*	Aphid
Strawberry necrotic shock virus	SNSV	*Ilarvirus SNSV*	*Bromoviridae*	Pollen
*Fragaria chiloensis* latent virus	FClLV	*Ilarvirus FCILV*	*Bromoviridae*	Thrips and pollen
Apple mosaic virus	ApMV	*Ilarvirus ApMV*	*Bromoviridae*	Pollen
Strawberry pseudo mild yellow edge virus	SPMYEV	*Carlavirus fragariae*	*Betaflexiviridae*	Not determined
Brassica yellows virus	BrYV	*Polerovirus TUYV*	*Solemoviridae*	Aphid
Cucumber mosaic virus	CMV	*Cucumovirus CMV*	*Bromoviridae*	Aphid

**Table 2:** Viruses infecting strawberry not presently classified by ICTV.

Virus name	Virus abbreviations	Description
Strawberry Kurdistan virus	SKV	Genome has 44–56% nucleotide identity to members of the genus *Crinivirus*, family *Closteroviridae*. Phylogenetic analysis indicates that SKV is a member of the *Crinivirus* group 2. The virus is whitefly-transmissible.
Strawberry virus A	StrVA	Discovered using high-throughput sequencing and possibly a novel Umbra-like Virus.
Strawberry associated virus 1	SaV1	SaV1 N and L genes share 32–57% and 38–64% amino acid sequence identity with those of nine reported cytorhabdoviruses. Probably a member of a novel species in the genus *Cytorhabdovirus*, family *Rhabdoviridae*.
*Fragaria vesca*-associated virus 1	FVaV-1	Genomic organization and sequence showed that this virus is related to members of the proposed insect-specific genus “Negevirus.”
*Fragaria chiloensis* cryptic virus	FClCV	Probably a member of the genus *Deltapartitivirus*. There is no known natural vector.

### 2.1 Strawberry vein banding virus (SVBV)

SVBV primarily infects cultivated and wild strawberry (*Fragaria vesca*). Symptoms include vein banding, leaf chlorosis, stunting, and reduced fruit yield (Cornuet and Morand, 1960; Ratti et al., 2009; Feng et al., 2016). Frequently, it co-occurs with tobacco necrosis virus (TNV) and cucumber mosaic virus (CMV), exacerbating symptom severity (Franova-Honetslegrova et al., 1999; Wang et al., 2000; Chen et al., 2014). SVBV is transmitted non-persistently by aphids in the field (e.g., *Chaetosiphon fragaefolii*) (Cornuet and Morand, 1960; Chen et al., 2014). Efficient transmission in the laboratory via particle bombardment (75–100% infection rates) and Agrobacterium-mediated inoculation have also been reported (Wang et al., 2000; Mahmoudpour, 2003). SVBV is known to occur in North America (USA, Canada), Europe (Italy, Czech Republic, Norway, Germany), Asia (China, Japan), and South America (Brazil) (Mraz et al., 1998; Ratti et al., 2009; Dickison et al., 2017; Ren et al., 2022). Divergent geographical clades in phylogenetic analysis of different open reading frames (ORFs), indicate that frequent recombination drives SVBV evolution, contributing to regional strain divergence (Mraz et al., 1998; Dickison et al., 2017; Ren et al., 2022).

SVBV belongs to the genus *Caulimovirus* within the family *Caulimoviridae*, sharing closest ancestry with cauliflower mosaic virus (CaMV), figwort mosaic virus, and carnation etched ring virus (Petrzik et al., 1998). Its genome consists of a circular double-stranded DNA (~7.8–7.9 kb) that encodes seven ORFs with functions analogous to those of CaMV. ORF I encodes P1 protein that is the movement protein (MP), regulating intracellular and intercellular movement during viral infection (Rui et al., 2022). SVBV P1 protein interacts with *F. vesca* chlorophyll-binding protein (FvLHC II-1 L), enhancing viral movement and accelerating infection (Xu et al., 2022). SVBV infection also regulates plant genes involved in pigment metabolism, and plant-pathogen defense pathways (Chen et al., 2016). ORF II and III may encode proteins associated with aphid infection and a DNA-binding protein, respectively. ORF IV and V encode the coat protein (CP) and a reverse transcriptase protein, respectively (Xu et al., 2022). P6, encoded by ORF VI, is a multifunctional protein that is an RNA silencing suppressor, interfering with systemic silencing signals and sequestering host translation factors (Feng et al., 2018; Li et al., 2018). ORF VII encodes an unknown protein.

### 2.2 Strawberry necrotic shock virus (SNSV)

SNSV is an economically significant pathogen affecting strawberry and *Rubus* species (e.g., blackberry, raspberry) in the U.S., Canada, Mexico, Japan and China (Tzanetakis et al., 2004b; Li and Yang, 2011; Silva-Rosales et al., 2013). Infected strawberry plants exhibit stunting, mild chlorosis, leaf reddening, and deformities (wrinkling, curling, mottling). Symptoms are often masked in mixed infections, complicating field diagnosis (Li and Yang, 2011; Silva-Rosales et al., 2013).

SNSV belongs to the genus *Ilarvirus* within the family *Bromoviridae.* Its genome consists of three positive-sense single-stranded RNA molecules (RNA1, RNA2, and RNA3), each encoding distinct functional proteins that coordinate viral replication, movement, and transmission. RNA1 (~3.3–3.5 kb) encodes 1a protein that contains methyltransferase (Met) and helicase (Hel) domains, essential for viral RNA replication initiation and 5′ cap modification, and that is the core component of the viral replication complex, driving genomic RNA synthesis (Tzanetakis et al., 2004b; Tzanetakis et al., 2010). RNA2 (~2.5–2.8 kb) encodes 2a and 2b proteins. 2a protein is the RNA-dependent RNA polymerase (RdRp), catalyzing viral RNA replication. 2b protein (present in some isolates) is the viral suppressor of RNA silencing (VSR), cooperating with the 1a protein to form the replicase complex for genome amplification. RNA3 (~2.2–2.4 kb) encodes a movement protein (MP) and coat protein (CP) (Tzanetakis et al., 2004b; Tzanetakis et al., 2010). Notably, the subgenomic RNA4, derived from the 3′ end of RNA3, often encodes additional CP copies. Viral RNAs have a 5′ cap structure that is probably methylated to protect RNA and enhance translation. The 3’ Untranslated region (UTR) of each RNA has conserved stem-loop structures for replication/packaging signals. Each RNA segment is independently packaged into virions; co-infection of all three is required for productive infection.

Initially, SNSV was misclassified as a strain of tobacco streak virus (TSV), but molecular studies showed that the complete RNA3 of SNSV (2,248 nucleotides) is 43 nucleotides longer than that of the TSV white clover isolate (TSV-WC) (Tzanetakis et al., 2004b). SNSV CP, a 669-nucleotide gene, is shorter than TSV-WC’s CP (714–717 nucleotides), while SNSV MP (897 nucleotides), is 27 nucleotides longer than TSV-WC’s MP. Furtherly, CP and MP sequences from 15 SNSV isolates form two distinct clusters sharing 95% intra-cluster amino acid identity but only 60–65% identity with TSV-WC (Tzanetakis et al., 2004b). This molecular divergence solidified SNSV’s status as a distinct virus within the genus *Ilarvirus* (family *Bromoviridae*) (Tzanetakis et al., 2004b; Tzanetakis et al., 2010; Li and Yang, 2011).

### 2.3 Strawberry mottle virus (SMoV)

SMoV infects both cultivated and wild strawberry. Experimental hosts include *Nicotiana occidentalis*, *Chenopodium quinoa*, and *F. vesca* (alpine strawberry), which exhibit symptoms such as leaf mottling, malformation, and stunting (Franova-Honetslegrova et al., 1998; Thompson et al., 2002). SMoV is primarily transmitted by aphids, notably *Chaetosiphon fragaefolii*, in a semi-persistent or non-persistent manner (Bonneau et al., 2019; Fan et al., 2022). It has been reported in Canada, the Czech Republic, Poland, the Netherlands, and China (Chen et al., 2014; Bonneau et al., 2019; Cieslinska, 2019). SMoV reduces yield by up to 50% in severe cases. High SMoV infection rates and yield reduction were linked to aphid vectors (38% of captured aphids tested positive) and wild *F. virginiana* reservoirs (Bonneau et al., 2019). Mixed infections with SMYEV and strawberry crinkle virus (SCV) synergistically amplify damage, with SCV alone showing greater detrimental effects on chlorophyll content and antioxidant enzyme activity (Thompson et al., 2002; Bonneau et al., 2019; Mozafari et al., 2022).

SMoV belongs to the genus *Sadwavirus* in the family *Secoviridae* and possesses a bipartite, positive-sense single-stranded RNA genome. RNA1 (7,036 nt) encodes a polyprotein that is cleaved for a helicase, VPg (viral genome-linked protein), a 3C-like cysteine protease, and an RNA-dependent RNA polymerase (RdRp) by the 3C-like cysteine protease. RNA2 (5,619 nt) encodes a polyprotein that is predicted to be cleaved into movement protein (MP), coat protein (CP), P28 and glutamic protease (Pro2Glu) by Pro2Glu (Mann et al., 2017; Mann et al., 2019). Phylogenetic analysis clusters SMoV with satsuma dwarf virus (SDV) and related viruses, though its aphid transmissibility distinguishes it from typical nepoviruses (Thompson et al., 2002; Mann et al., 2017; Mann et al., 2019). P28 and Pro2Glu are recognized as the VSRs of SMoV (Fan et al., 2022). These proteins inhibit local and systemic RNA silencing triggered by single-stranded GFP RNA. P28 exacerbates symptoms of co-infecting viruses like potato virus X (PVX) by enhancing viral accumulation (Fan et al., 2022).

### 2.4 Strawberry latent ringspot virus (SLRSV)

Strawberry latent ringspot virus (SLRSV) infects a broad range of plants, including strawberry, blackberry (*Rubus* spp.), pepino (*Solanum muricatum*), impatiens (*Impatiens walleriana*), and ornamental species like *Tibouchina* spp. and *Anemone* x *hybrida*. Infections are often latent, complicating detection (Martin et al., 2004; Tang et al., 2013). SLRSV is transmitted by dagger nematodes (*Xiphinema* spp.) in a persistent manner. Mechanical transmission to herbaceous hosts (e.g., *Chenopodium quinoa*) is also documented (Martin et al., 2004). SLRSV has been reported from Europe, New Zealand and North America (Martin et al., 2004; Tang et al., 2013; Dullemans et al., 2020). In Europe, it is historically widespread, with significant diversity reported in isolates (Dullemans et al., 2020). In New Zealand, SLRSV has been identified in multiple hosts. Phylogenetic analysis revealed two distinct strains: one infecting blackberry/impatiens and another in pepino/tibouchina, both divergent from global isolates (Tang et al., 2013). In North America, it has been detected in strawberries in California (17% infection rate) and British Columbia (4%). Isolates share 84% nucleotide identity with European strains (Martin et al., 2004).

SLRSV is a member of the family *Secoviridae*, with recent proposals to classify it under a novel genus *Stralarivirus*, alongside lychnis mottle virus and two SLRSV subgroups (SLRSV-A and SLRSV-B) (Dullemans et al., 2020). It has a bipartite RNA Genome. RNA1 encodes a polyprotein containing protease-polymerase (Pro-Pol) domains, critical for replication. Amino acid sequences in this region are highly conserved across isolates, despite nucleotide-level variability (Dullemans et al., 2020). RNA2 encodes a polyprotein processed into movement and capsid proteins (CPs). The large and small CPs exhibit lower amino acid conservation compared to Pro-Pol, correlating with serological diversity among isolates (Kreiah et al., 1994; Dullemans et al., 2020). RNA2 sequences show limited homology with other nepoviruses or comoviruses, supporting its classification within a distinct genus (Kreiah et al., 1994). Some SLRSV isolates associate with a satellite RNA (~1.2 kb) containing a long ORF encoding a 36.5 kDa protein. This satellite RNA lacks homology with known sequences in public databases, suggesting a unique evolutionary origin (Kreiah et al., 1993).

### 2.5 Strawberry mild yellow edge virus (SMYEV)

SMYEV infects the cultivated strawberry and wild relatives. In laboratory tests, it infects *Rubus rosifolius* and *Chenopodium quinoa*, which develop localized or systemic infections (Jelkmann et al., 1990; Lamprecht and Jelkmann, 1997; Franova, 2001). SMYEV is primarily transmitted by aphids (e.g., *Chaetosiphon fragaefolii*) in a non-persistent manner. Strain-specific transmissibility is noted; for example, isolate D74 is aphid-transmissible, while MY18 is not (Thompson and Jelkmann, 2004). SMYEV is widespread in Europe, North America (Canada, Mexico), and Asia (China). In Canada, a 2012–2013 survey identified 85 SMYEV CP haplotypes across four provinces, with mixed infections in 68% of samples (Xiang et al., 2020). SMYEV often causes asymptomatic infections in strawberries, complicating detection. Severe symptoms including leaf yellowing, stunting and deformed fruits, occur in mixed infections with viruses like SMoV or criniviruses (Franova, 2001; Silva-Rosales et al., 2013; Chen et al., 2014).

SMYEV, belonging to the genus *Potexvirus* (family *Alphaflexiviridae*), is a single-stranded, positive-sense RNA virus. The SMYEV genome (~6 kb) contains six ORFs encoding proteins involved in replication (ORF1: RNA-dependent RNA polymerase), movement (triple gene block proteins, TGB1-3), and encapsidation (coat protein, CP). Unique features include overlapping TGB3 and CP coding regions and a non-AUG initiation codon for ORF2 (TGB1), distinguishing it from other potexviruses (Jelkmann et al., 1992; Thompson and Jelkmann, 2004).

SMYEV isolates exhibit significant genetic divergence. Canadian variants form unique subclades (88% of haplotypes), likely originating from external sources but adapting locally through mutations (Xiang et al., 2020). Phylogenetic analysis of the CP gene divides SMYEV isolates into three major clades: Strain I (type-D74, predominant in Europe), Strain II (type-9Redland), and Strain III (type-MY18, non-transmissible via aphids). Canadian isolates form a distinct, highly divergent population, suggesting adaptive evolution (Thompson and Jelkmann, 2004; Xiang et al., 2020). Meanwhile, overlapping ORFs and conserved motifs (e.g., stem-loop structures in untranslated regions) suggest evolutionary strategies to maintain functionality despite genomic variability (Thompson and Jelkmann, 2004).

### 2.6 Strawberry pallidosis-associated virus (SPaV)

SPaV is prevalent in major strawberry-producing regions, including California, Oregon, and the Mid-Atlantic States. Infection rates reached 90% in symptomatic plants in areas with high whitefly populations (Tzanetakis et al., 2003; Tzanetakis et al., 2006). It primarily infects cultivated and wild strawberry. Experimental hosts include *Nicotiana benthamiana* (Tzanetakis et al., 2003). The greenhouse whitefly (*Trialeurodes vaporariorum*) is the confirmed vector, transmitting SPaV in a semi-persistent manner. Seed (achene) and pollen transmission were investigated but found to be negligible (Tzanetakis et al., 2006). Wild *F. virginiana* patches near commercial fields act as significant viral reservoirs (Bonneau et al., 2019). Pallidosis disease manifests as marginal leaf chlorosis, epinasty, and plant decline. High infection rates (e.g., 90% in California) correlate with reduced vigor and yield, particularly in regions with intensive whitefly activity (Tzanetakis et al., 2006). Symptoms are often exacerbated in mixed infections with viruses like SMoV or SCV (Silva-Rosales et al., 2013; Bonneau et al., 2019).

SPaV is a member of the genus *Crinivirus* (family *Closteroviridae*), characterized by a bipartite, single-stranded, positive-sense RNA genome. RNA1 (8,067 nt) encodes ORF1a (a multifunctional protein with papain-like protease, methyltransferase, and RNA helicase domains), ORF1b (RNA-dependent RNA polymerase, expressed via + 1 ribosomal frameshift), and a small transmembrane protein at the 3′ end. RNA2 (7,979 nt) contains eight ORFs encoding proteins homologous to other criniviruses, including a minor coat protein (CPm) of ~80 kDa, the largest structural protein reported in *Closteroviridae*. SPaV is closely related to Abutilon yellows virus and Beet pseudo-yellows virus (BPYV), with which it shares ~56% nucleotide identity in the 3′ non-translated regions (Tzanetakis et al., 2005; Tzanetakis et al., 2006). SPaV frequently coexists with BPYV, causing identical pallidosis symptoms (leaf chlorosis, epinasty). In California USA, co-infection rates reached 60% in symptomatic plants (Tzanetakis et al., 2003; Tzanetakis et al., 2006).

### 2.7 Strawberry polerovirus 1 (SPV-1)

SPV-1 has been reported across multiple continents, highlighting its expanding range. In North America, it was first identified in Canada in 2015 and later detected in the U. S., where it contributes to strawberry decline (Xiang et al., 2015; Kwak et al., 2022). In Europe, it has been reported in the Czech Republic (35% prevalence in surveyed plants) and Italy, marking its spread to Mediterranean regions (Franova et al., 2021; Cultrona et al., 2024). It has also been detected in Argentina and Nepal, the latter representing its first report in Asia (Kwak et al., 2022). Aphids are the main transmission agents of SPV-1. Confirmed vectors include *Chaetosiphon fragaefolii* (strawberry aphid) and *Aphis gossypii* (cotton aphid). Transmission requires ≥4 h of acquisition access and ≥1 day of inoculation access (Franova et al., 2021). SPV-1 has been detected in non-aphid invertebrates and aphid honeydew, though their role in transmission remains unclear (Franova et al., 2021). SPV-1 is often associated with strawberry decline syndrome, characterized by severe dwarfing, leaf cupping, chlorotic spotting, and plant death. However, there is a report that 35% of infected plants were asymptomatic (Franova et al., 2021; Cultrona et al., 2024). SPV-1 frequently co-occurs with viruses like SMoV and SMYEV, potentially exacerbating disease severity (Xiang et al., 2015; Cultrona et al., 2024).

SPV-1 is a member of the genus *Polerovirus* (family *Solemoviridae*), characterized by a monopartite, single-stranded, positive-sense RNA genome. The SPV-1 genome (~5.9–6.0 kb) has the six ORFs typical of poleroviruses, including a P1-P2 fusion protein (involved in viral replication), a coat protein (CP), and a read-through domain (RTD) essential for aphid transmission (Kwak et al., 2022; Cultrona et al., 2024). Phylogenetic analyses reveal at least two distinct clades among SPV-1 isolates. Recombination events have been identified in Czech isolates, suggesting evolutionary adaptability (Franova et al., 2021; Kwak et al., 2022). SPV-1 isolates from Canada, Argentina, and Nepal have high nucleotide identity (97–99%), indicating global genetic conservation (Kwak et al., 2022; Cultrona et al., 2024).

## 3 Strawberry-infecting virus-host interactions

**Figure 2:**
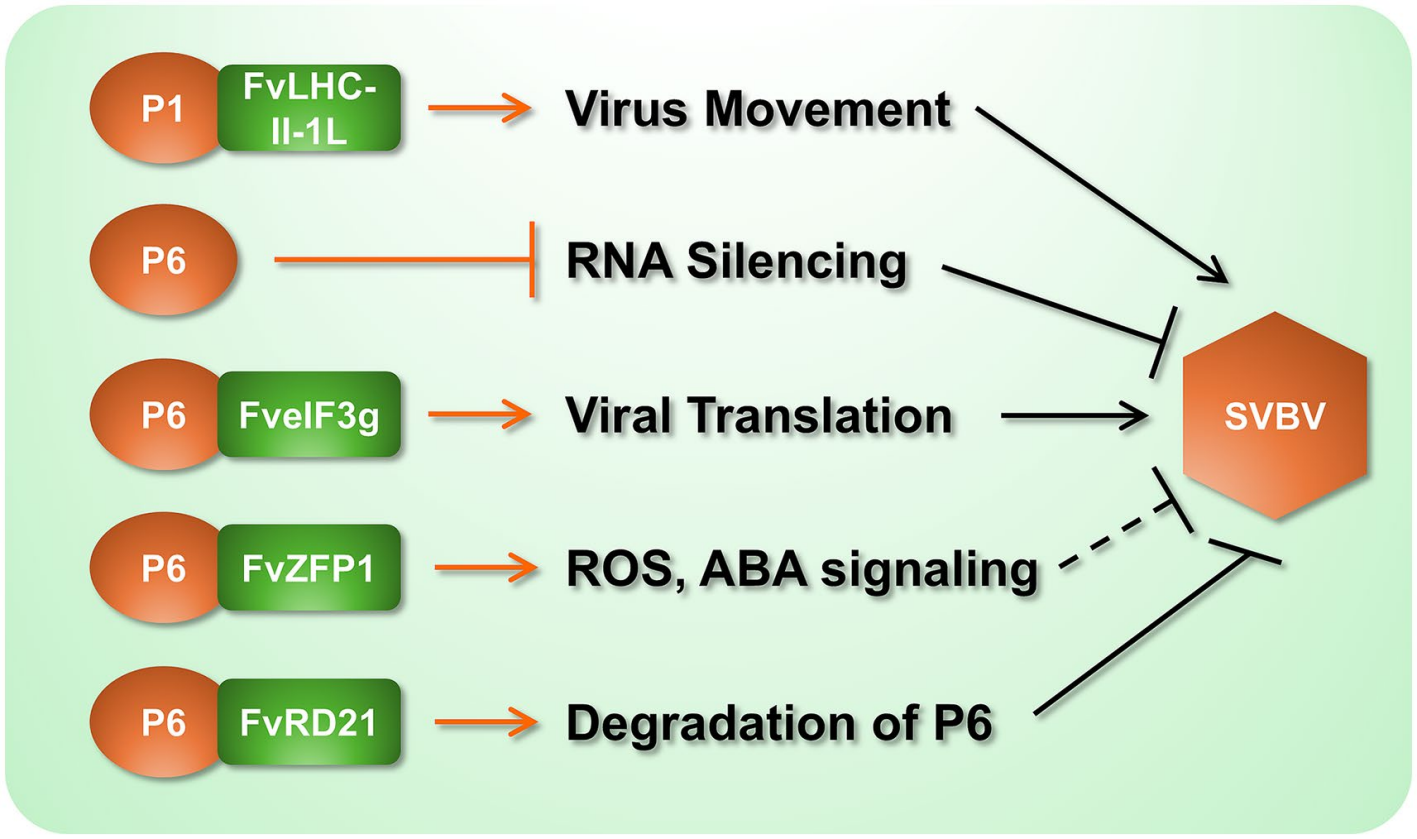
Molecular interactions between SVBV and strawberry components. Black arrows indicate proviral effects that facilitate SVBV infection. Black blunt-ended lines denote the responses that suppress viral infection. It should be noted that the function of FvZFP1 was analyzed by its heterologous expression in *N. benthamiana* to show resistance to TMV, and its effect on SVBV remains to be verified (indicated by the dotted line in the figure).

Investigations of virus-host interactions elucidate molecular strategies through which pathogens co-opt cellular machinery to establish infection, while revealing evolutionary arms races that shape host defense countermeasures. In strawberry, seminal advances derive from studies of SVBV, where viral movement protein P1 and silencing suppressor P6 critically subvert host functions (Figure 2). Xu et al. (2022) demonstrated that SVBV P1 directly interacts with the chloroplast-localized *F. vesca* light-harvesting complex II protein (FvLHC II-1 L), a core component of photosynthetic electron transport. This interaction potentiates viral movement: P1 recruits FvLHC II-1 L to rescue cell-to-cell trafficking in movement-deficient PVXΔP25 and systemic spread in CMVΔMP. Crucially, transient overexpression of FvLHC II-1 L accelerated SVBV infection kinetics in *F. vesca*, confirming that viral exploitation of photosynthetic machinery enhances pathogenesis (Xu et al., 2022). Concurrently, SVBV P6 (ORF VI product) functions as a multifunctional trans-activator, forming nuclear-cytoplasmic granules that enhance viral mRNA translation. Notably, this host factor disrupts P6 granule assembly and attenuates its translational activation capacity—revealing a potent host counter-strategy (Li et al., 2018). Recently, a papain-like cysteine protease (PLCP) in *Fragaria vesca*, FvRD21, has been shown to interact with P6, inducing its autophagic degradation and so destroying its VSR function and conferring plant resistance to SVBV (Yang et al., 2025).

Transcriptomic profiling of SVBV-infected *F. vesca* corroborates these manipulative mechanisms, identifying 517 differentially expressed genes enriched in pigment metabolism, photosynthesis, and plant-pathogen interaction pathways (Chen et al., 2016). Countering viral aggression, the host deploys zinc finger protein FvZFP1 as a defense orchestrator (Rui et al., 2022). Heterologous expression in *N. benthamiana* confirmed that FvZFP1 elevates ROS production and activates salicylic acid (SA) pathways, conferring resistance against tobacco mosaic virus and *Pseudomonas syringae*. FvZFP1 additionally enhances abiotic stress tolerance through ABA-mediated signaling and antioxidant modulation (SOD/MDA dynamics), establishing its role as a hub for biotic-abiotic stress cross-talk (Rui et al., 2022).

## 4 Diagnostic technologies for strawberry-infecting viruses

In the early stages of research into strawberry-infecting viruses, transmission electron microscopy (TEM) and serological methods were widely employed for virus detection and identification (Jelkmann et al., 1990; Franova-Honetslegrova et al., 1998; Franova, 2001; Martin et al., 2004; Tang et al., 2013; Cieslinska, 2019; Dullemans et al., 2020). Compared to TEM, serological techniques offer advantages such as ease of operation and lower cost, and in certain scenarios, enable high-throughput screening—for instance, through enzyme-linked immunosorbent assay (ELISA). As a result, such assays have been extensively utilized in virological studies (Martin and Tzanetakis, 2006). However, with the rapid advances in molecular biology, the limitations of serology-based approaches have become increasingly evident. For example, polyclonal antibodies often lack the specificity required to distinguish among different virus isolates (Jelkmann et al., 1990; Franova, 2001; Tzanetakis and Martin, 2004), and low viral titers can lead to false-negative results, highlighting the suboptimal sensitivity of these methods (Mraz et al., 1997; Tzanetakis et al., 2004a). This review focuses on the molecular detection techniques that are currently in widespread use or under active development and discusses their potential applications in the detection of strawberry-infecting viruses (Table 3).

**Table 3:** Developed technologies for detecting strawberry-infecting viruses.

Technology	Detection target viruses
TEM	SVBV, CMV, SMYEV, SLRSV, TNV
ELISA	SMYEV, CMV, SCV, SVBV, TNV, SMoV, ApMV
PCR/RT-PCR	All viruses infecting strawberry
Multiplex PCR	SPaV, SMYEV, SVBV, SMoV, SPV-1, SCrV-4, StrV-2, SLRSV
qPCR	StrVA, FVaV-1, BrYV, SNSV
Co-PCR	SLRSV, CMV
HTS	SMoV, SVBV, SPaV, StrV-1, BrYV, SPV-1, SMYEV, SCV, SNSV, BPYV, ToRSV, ApMV, SCFaV, FClLV, FClCV
RT-RPA-LFS	SMoV, SMYEV
CRISPR-Cas-based	StrV-3

### 4.1 PCR-based methods

Polymerase chain reaction (PCR) and its derivatives amplify virus-specific nucleic acid sequences, enabling highly sensitive and specific detection. These techniques have become fundamental tools in strawberry-infecting virus diagnostics due to their accuracy, adaptability, and broad applicability (Martin et al., 2004; Li and Yang, 2011; Silva-Rosales et al., 2013; Chen et al., 2014; Kwak et al., 2022; Cultrona et al., 2024). In addition to conventional PCR and RT-PCR, multiplex PCR has gained prominence for its ability to simultaneously detect multiple viruses in a single reaction, thereby significantly reducing time and cost. Wang et al. developed a multiplex PCR assay capable of detecting six strawberry-infecting viruses, including SMYEV, SVBV, SMoV, SPV-1, SPaV, and strawberry crinivirus 4 (SCrV-4) using optimized primer sets (Wang et al., 2024). To ensure RNA integrity and validate amplification efficiency, Thompson et al. incorporated a plant-derived internal control, such as malate dehydrogenase mRNA, into their assay system simultaneously detecting SCV, SMYEV, SMoV and SVBV (Thompson et al., 2003). The upper detection limit for the four viruses was at an extract dilution of 1/200 (Thompson et al., 2003). Bertolini et al. (2003) designed a multiplex nested RT-PCR that enabled, for the first time, the sensitive and simultaneous detection of RNA and DNA targets from four plant viruses (cucumber mosaic virus, cherry leaf roll virus, SLRSV and arabis mosaic virus) and a bacterial pathogen (*Pseudomonas savastanoi* pv. *savastanoi*), a procedure which had an 8.1% increase in sensitivity compared to conventional RT-PCR methods.

qPCR, particularly TaqMan-based assays, quantifies viral loads with high sensitivity, ideal for detection of low-titer viruses. Zhao et al. established a TaqMan RT-qPCR assay for BrYV in strawberries, achieving a detection limit of 100 fg (100x more sensitive than RT-PCR) (Zhao et al., 2022). Thekke Veetil et al. (2016) developed a qPCR assay for SNSV, that is at least 100 times more sensitive than conventional RT-PCR.

A Cooperative Amplification (Co-PCR) method was developed for sensitive plant virus detection, utilizing a tetraprimer system that simultaneously reverse transcribes two overlapping target fragments, generates four amplicons via nested primer pairs, and cooperatively amplifies the longest product (Olmos et al., 2002). This technique had a 100-fold higher sensitivity than conventional RT-PCR (comparable to nested RT-PCR) when detecting five plant RNA viruses (cherry leaf roll virus, SLRSV, CMV, plum pox virus, and citrus tristeza virus), and provided a colorimetric readout for diagnostic applications (Olmos et al., 2002).

### 4.2 High-throughput sequencing (HTS)

HTS has transformed strawberry-infecting virus research by enabling sensitive detection, novel virus discovery, and population diversity analyses (Roossinck et al., 2015; Wu et al., 2015; Roossinck, 2017; Villamor et al., 2019). Metatranscriptomic approaches using rRNA-depleted samples can detect viruses like SMoV at concentrations as low as 10 copies/μL, matching qPCR sensitivity (Nunes-Leite et al., 2024). HTS has identified 14 known viruses plus novel variants in strawberry germplasms, including emerging pathogens like tomato brown rugose fruit virus, while also characterizing viral quasispecies through complete genome sequencing of multiple isolates (Diaz-Lara et al., 2021). Moreover, the technology proved critical for discovering novel viruses. Near-complete genomes of prunus virus I (phylogenetically related to SNSV) and SPV-1 were achieved from 31 million sequencing reads (Orfanidou et al., 2021; Kwak et al., 2022; Cultrona et al., 2024). Rajamaki et al. identified rubus yellow net virus (RYNV) in Finnish raspberry germplasms, suggesting that endogenous viruses may evade traditional assays (Rajamaki et al., 2019). Franova et al. found a novel rhabdovirus, tentatively named strawberry virus 1 (StrV-1), that infects *F. ananassa* and *F. vesca* plants (Franova et al., 2019). These applications demonstrate the value of HTS for both fundamental virology and applied diagnostics of strawberry-infecting virus (Dullemans et al., 2020; Diaz-Lara et al., 2021; Orfanidou et al., 2021).

### 4.3 Isothermal nucleic acid amplification-based lateral flow testing (INAA-LFT)

INAA-LFT has emerged as robust technique for the on-site rapid detection of plant viruses (Song et al., 2024). Under isothermal conditions, nucleic acid amplification techniques such as loop-mediated isothermal amplification (LAMP), recombinase polymerase amplification (RPA), or recombinase-mediated chain replacement nucleic acid amplification (RAA) are employed to amplify target nucleic acid fragments from pathogenic DNA or RNA (Boonham et al., 2014). Efficient amplification of the target nucleic acid fragments is critical for the highly sensitive detection of INAA-LFTs. Reverse Transcription Recombinase Polymerase Amplification (RT-RPA) performs isothermal amplification (40°C) within 15 min, combined with LFS for visual readouts of SMoV (Zou et al., 2022b). The detection limit of the optimized assay for SMoV was 500 fg of RNA. It was used to detect suspected samples from the field, and showed good concordance with RT-PCR results in the lab, indicating its potential use for rapid on-site detection of SMoV (Zou et al., 2022b). A similar assay for detection of SMYEV had a sensitivity 100 times higher than that of RT-PCR (10 pg/μL) and with the same accuracy (Zou et al., 2022a).

## 5 Prospect

### 5.1 Biology of strawberry-infecting viruses

Current research on strawberry-infecting viruses has significantly advanced our understanding of virus diversity, epidemiology, and detection; however, several critical knowledge gaps remain that warrant further investigation. For example, epidemiological studies are still limited, particularly concerning the global distribution and prevalence of viruses such as SNSV and SPaV in understudied regions, including Africa and Southeast Asia (Kwak et al., 2022). Historically, virus surveys have relied on manual and labor-intensive field inspections, which constrained large-scale surveillance. In contrast, recent developments in artificial intelligence (AI)-based pest and disease recognition systems, including image-based and hyperspectral analysis, offer promising opportunities for rapid, scalable monitoring (Tannous et al., 2023; Zhang et al., 2024; Bai et al., 2025). While these technologies have yet to be widely applied to strawberry-infecting virus surveillance, especially under field conditions, their integration could greatly enhance studies on virus distribution, biology, and early-warning systems.

A distinguishing feature of strawberry virology is the high frequency of mixed infections, typically involving viruses from distinct genera and species (Franova, 2001; Thompson et al., 2002; Tzanetakis et al., 2003; Tzanetakis et al., 2006; Silva-Rosales et al., 2013; Chen et al., 2014; Bonneau et al., 2019; Mozafari et al., 2022). These co-infections, such as those involving SMYEV and SCV, are thought to produce synergistic effects that exacerbate disease symptoms and yield losses (Thompson et al., 2002; Bonneau et al., 2019; Mozafari et al., 2022). However, the mechanisms underlying these synergistic interactions remain poorly understood. It is unclear whether synergism influences virus accumulation, transmission efficiency by vectors, or host pathogenic responses. Additionally, potential antagonistic interactions between co-infecting viruses have not been thoroughly explored. Addressing these questions is essential for developing effective management strategies in multi-virus contexts.

### 5.2 Molecular biology of strawberry-infecting viruses

Recent advances in plant virology over the past decade have significantly enhanced our understanding of viral gene functions and the plant-virus interactions (Wu et al., 2024). However, these insights have yet to be fully translated to strawberry-infecting virus systems. Many key viral proteins in strawberry-infecting viruses remain functionally uncharacterized. For instance, the function of the ORF VII protein encoded by SVBV is still unknown, and while the HSP70 homolog encoded by SPaV is presumed to be involved in replication complex formation, its exact role is unclear. Similarly, the putative protease and polymerase domains encoded by RNA2 of SLRSV have not been experimentally validated. Furthermore, the functional significance of satellite RNAs associated with SLRSV and SMYEV in modulating disease severity and their potential role in cross-protection remain unexplored. Clarifying the roles of key viral proteins will be pivotal to understanding symptom development and virus evolution under natural mixed infection scenarios (Jelkmann et al., 1992; Thompson and Jelkmann, 2004; Xu et al., 2022).

Advances in general plant virology over the past decade have revealed the central role of viral suppressors of RNA silencing and virus-derived small RNAs (vsRNAs) in modulating host defense responses (Wu et al., 2024). Numerous viral proteins have been shown to interfere with host RNA silencing pathways or manipulate host immunity to facilitate infection (Burgyan and Havelda, 2011; Jin et al., 2021; Pan et al., 2021; Zhao and Li, 2021; Sharma and Prasad, 2025). Moreover, vsRNAs have been implicated in the regulation of host gene expression during infection (Yan et al., 2010; Shi et al., 2016; Ramesh et al., 2020; Zhang et al., 2021). In comparison to well-studied viral systems such as CMV or tomato yellow leaf curl virus (TYLCV), where RNA silencing suppressors have been extensively characterized, strawberry-infecting viruses lag behind. For example, the Pro2Glu/P28 protein of SMoV has been suggested as a silencing suppressor, yet its precise function and molecular mechanisms remain unverified (Fan et al., 2022). This lack of fundamental knowledge regarding the interactions between strawberry-infecting viruses and host immune responses underscores an urgent need for in-depth molecular studies.

### 5.3 Detection of strawberry-infecting viruses

In the realm of diagnostics, strawberry virology has benefited from significant technological progress. Molecular tools such as ELISA, RT-PCR, qPCR, and HTS have dramatically improved the accuracy and scope of virus detection. Nonetheless, reliance on a single method often risks false negatives or incomplete virus identification. As such, a multilayered detection strategy is recommended (Villamor et al., 2022). For initial screening, serological methods like ELISA or dot-blot hybridization can be employed, followed by nucleic acid-based confirmation using RT-PCR or qPCR (Mraz et al., 1997). HTS offers powerful capabilities for novel virus discovery and virome profiling, with PCR-based validation confirming virus prevalence (Diaz-Lara et al., 2021). Field-deployable methods such as reverse transcription–recombinase polymerase amplification coupled with lateral flow assays (RT-RPA-LF) provide rapid preliminary results, which can be verified by laboratory-based qPCR (Zou et al., 2022a; Zou et al., 2022b). Recent studies have demonstrated the utility of multiplexed and nested PCR to enhance detection sensitivity and specificity (Bertolini et al., 2003; Diaz-Lara et al., 2021).

Notably, the development of integrated INAA-LFT systems enables highly sensitive, rapid on-site detection of viruses, even before visible symptoms emerge (Song et al., 2024). However, the current application of INAA-LFT for strawberry-infecting viruses remains limited in scope (Cao et al., 2020; Song et al., 2024; Cao et al., 2025). Expanding these systems to detect a broader spectrum of strawberry-infecting viruses is imperative. Moreover, the practicality of these tests depends on the simplicity and speed of sample processing. Many field settings lack access to specialized equipment for nucleic acid extraction or reverse transcription. Encouragingly, simplified protocols integrating cell lysis, reverse transcription, and isothermal amplification have been developed for other plant viruses and may serve as a model for similar systems in strawberry-infecting virus diagnostics. Finally, integrating AI-assisted analysis—such as colorimetric interpretation of lateral flow results—can further streamline diagnostics, reduce human error, and facilitate real-time decision-making in virus management. Moving forward, the combination of AI-powered tools, portable molecular diagnostics, and enhanced mechanistic understanding of virus-host interactions will be key to advancing strawberry virology and securing global strawberry production (Song et al., 2024).
